# Transcriptome Analysis Reveals Significant Differences in Gene Expression of Malignant Pheochromocytoma or Paraganglioma

**DOI:** 10.1155/2019/7014240

**Published:** 2019-05-08

**Authors:** Yong Joon Suh, Jung Ho Park, Sanchir-Erdene Bilegsaikhan, Dong Jin Lee

**Affiliations:** ^1^Department of Breast and Endocrine Surgery, Hallym University Sacred Heart Hospital, Anyang 14068, Republic of Korea; ^2^Department of Otolaryngology-Head and Neck Surgery, Hallym University College of Medicine, Seoul 07441, Republic of Korea

## Abstract

Prediction of malignant behavior of pheochromocytoma (PC) or paraganglioma (PG) is of limited value. The Cancer Genome Atlas (TCGA) and the French ‘Cortico et Médullosurrénale: les Tumeurs Endocrines' (COMETE) network in Paris (France) facilitate accurate differentiation of malignant PC/PG based on genetic information. Therefore, the objective of this transcriptome analysis is to identify the prognostic genes underlying the differentiation of malignant PC/PG in the TCGA and COMETE databases. TCGA carries data pertaining to multigenomic analysis of 173 PC/PG surgical resection samples while the COMETE cohort contains data involving 188 PC/PG surgical resection samples. Clinical information and mRNA expression datasets were downloaded from TCGA and COMETE databases. Based on eligibility criteria, 58 of 173 PC/PG samples in TCGA and 171 of 188 PC/PG samples collected by the COMETE network were selected. Using Ingenuity Pathway Analysis, the mRNA expression of malignant and benign PC/PG was compared. The 58 samples in TCGA included 11 malignant and 47 benign cases. Among the 171 samples obtained from the COMETE cohort, 19 were malignant and 152 were benign. A comparative analysis of the mRNA expression data of the two databases revealed that 11 up/downregulated pathways involved in malignant PC/PG were related to cancer signaling, metabolic alteration, and prominent mitosis, whereas 6 upregulated genes and 1 downregulated gene were significantly enriched in the functional annotation pathways. The TCGA and COMETE databases showed differences in mRNA expression associated with malignant and benign PC/PG. Improved recognition of prognostic genes facilitates the diagnosis and treatment of PC/PG.

## 1. Introduction

Pheochromocytoma (PC) is a catecholamine-secreting neuroendocrine neoplasm originating in the adrenal medulla [1]. Paraganglioma (PG) is a catecholamine-producing neuroendocrine neoplasm developing in the extra-adrenal chromaffin tissue of sympathetic ganglia. Nearly 15–20% of PC/PG originated in extra-adrenal chromaffin tissues whereas 80–85% of PC/PG develops from adrenal medulla. The annual incidence of PC/PG varies between 2 and 8 per million. In the population, the prevalence of PC/PG ranges from 1:6,500 to 1:2,500, respectively [2, 3]. Clinical manifestations include hypertension, tachycardia, headache, diaphoresis, and anxiety [4].

Diagnostic tests for PC/PG include imaging, biochemical evaluation, and histopathology, in addition to genetic testing [5–7]. Patients with PC/PG are managed via surgery, medical treatment, chemotherapy, targeted radiation therapy using ^131^I-MIBG, embolization, cryoablation, targeted molecular therapy, and radiofrequency ablation [8]. Due to the diagnostic uncertainty, management usually entails vigilant monitoring for metastasis in PC/PG.

The tumor conforms to “the rule of 10s” and 10% of PC/PG is considered malignant [9, 10]. However, the malignancy rate exceeds 10% in patients with extra-adrenal disease [11]. Malignant PC/PG is associated with a 5-year survival rate of around 50% [12, 13]. The patients' long-term survival has yet to be improved [14].

Histological analysis cannot be used to predict the malignant or benign behavior of PC/PG [15]. According to the 8th edition of the American Joint Committee on Cancer (AJCC) staging system, distant metastasis is the only distinctive feature of malignant PC/PG. Therefore, this diagnostic limitation restricts therapeutic planning. Accurate diagnosis is directly linked to successful management. Institutions have struggled to define molecular markers for malignant PC/PG [16, 17]. Studies to discover robust predictors of malignancy are still ongoing [18, 19].

Recent molecular data obtained from The Cancer Genome Atlas (TCGA) and the French ‘Cortico et Médullosurrénale: les Tumeurs Endocrines' (COMETE) cohort are available in the public domain [20–22]. The databases describe tumors including mRNA expression and clinical information. The TCGA and COMETE databases may be used for transcriptome analysis of malignant PC/PG. The aim of the present study was to identify potential biomarkers based on the mRNA expression profile of TCGA and COMETE databases for diagnosis of malignant PC/PG.

## 2. Materials and Methods

### 2.1. Study Design

**Figure 1:**
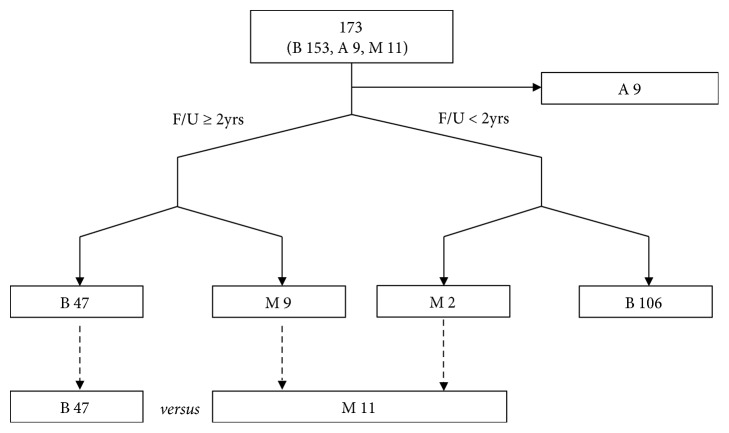
Flow diagram outlining enrollment protocol in TCGA. PC: pheochromocytoma; PG: paraganglioma; M: malignant; A: aggressive; B: benign; F/U: follow-up.

**Figure 2:**
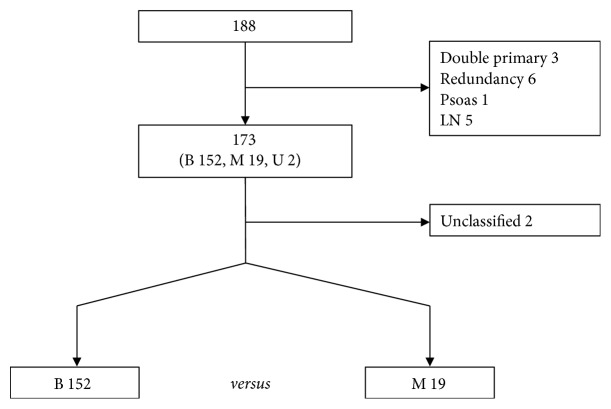
Flow diagram outlining the enrollment protocol in COMETE cohort. PC: pheochromocytoma; PG: paraganglioma; M: malignant; B: benign; U: unclassified; LN: lymph node.

Accessible data obtained from TCGA and COMETE cohort were used to compare malignant and benign PC/PG cases. The TCGA data contains multigenomic analysis of 173 PC/PG surgically resected samples. The initial pathological diagnosis was conducted from 1988 to 2013. Clinical information and gene expression dataset (20,531 genes) of RNA-sequencing mRNA Fragments Per Kilobase Million (FPKM) were downloaded from the TCGA database. The dataset was assembled into a table. Only samples with TCGA type code 01 (primary solid tumor) were included (Figure 1). Benign samples were included only if the follow-up exceeded two years. However, malignant samples were included even if they were metastasized within two years. Aggressive samples with local invasion or metastatic lymph nodes were excluded due to uncertain behavior. A total of 58 samples met the inclusion criteria. The 58 PC/PG samples included 11 malignant and 47 benign cases. Clinical characteristics and mRNA expression were reorganized according to tumor behavior and compared with each other. The COMETE cohort carried multiomic analytical data pertaining to 188 PC/PG surgically resected samples from the French COMETE Network. Cases were recruited from 1993 to 2008. Clinical information and expression dataset (39,534 probes) of mRNA transcripts were downloaded from the COMETE cohort. Only primary tumor samples were included (Figure 2). A total of 171 samples contained accessible genomic data and corresponding clinical information. The 171 samples included 19 malignant and 152 benign cases. Clinical characteristics and mRNA expression were reorganized according to behavior and compared. The gene expression was interpreted by extracting differentially expressed genes (DEGs) from TCGA and COMETE cohort. Commonly enriched genes were searched via Ingenuity Pathway Analysis (IPA). The identified genes were validated using the Dutch cohort (GSE67066) [23].

### 2.2. Definition

Metastasis was defined as the presence of chromaffin tissue at nonchromaffin sites distant from the primary tumor. Malignant or benign behavior was used to define metastasis or lack thereof, respectively. A zero value was filtered for the analysis of mRNA. To identify DEGs, a false discovery rate < 0.05 and a log_2_ fold change ≥ 1.5 were set as the threshold values. The functional enrichment of genes was analyzed based on Gene Ontology.

### 2.3. Statistics

Malignant and benign PC/PG groups were compared using Chi-squared test or Fisher's exact test for categorical data and Student's t-test or Mann-Whitney* U* test for continuous data without normal distribution. In two-tailed tests, a* p* value below 0.05 was considered statistical significant. Statistical analysis was performed using R 3.4.1 (R Foundation for Statistical Computing, Vienna, Austria), GSEA 2.1.0 (Broad Institute, MA, USA), and IPA (Ingenuity Systems, CA, USA). The overall survival rate was estimated according to the degree of gene expression using the UALCAN platform [24].

## 3. Results

### 3.1. Clinical Characteristics

**Figure 3:**
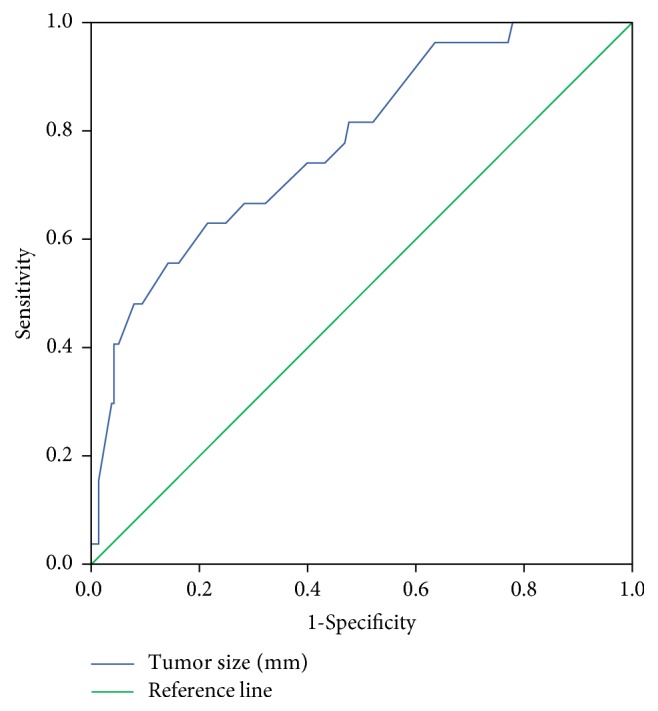
According to the ROC curves, the size of 54.5 mm yielded the highest accuracy in TCGA and COMETE cohort (AUC = 0.778, sensitivity = 66.7%, specificity = 61.6%, and* p* < 0.001). ROC: receiver operating characteristic; AUC: area under the curve.

A total of 58 patients with PC/PG from TCGA were included in the current study. They included 11 patients with metastasis assigned to the malignant PC/PG group. Among 171 patients with PC/PG derived from COMETE cohort, there were 19 patients with malignant PC/PG. TCGA and COMETE cohort showed no age or gender difference in malignant and benign manifestations (Table 1). The mean follow-up periods were 2460.2 ± 2658.9 days and 1617.6 ± 867.7 days in malignant and benign PC/PG, respectively (*p* = 0.320). The proportion of cases diagnosed with paraganglioma was significantly higher among cases of malignant PC/PG (*p* < 0.001). The size was also significantly greater in malignant PC/PG (*p* < 0.001). The optimal cut-off value was calculated to identify malignancy. According to the ROC curves, the highest accuracy was obtained with a size of 54.5 mm (AUC = 0.778, sensitivity = 66.7%, specificity = 61.6%, and* p* < 0.001) (Figure 3). Dopamine secretion was more frequent in malignant PC/PG, whereas metanephrine secretion was only detected in benign cases of PC/PG (Table 2).

### 3.2. mRNA Analysis

**Figure 4:**
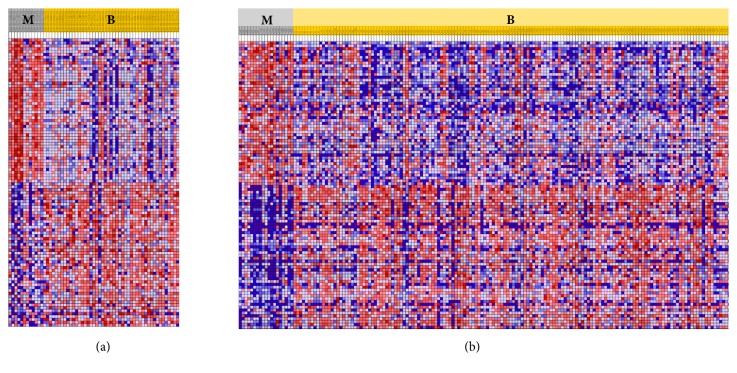
Gene Set Enrichment Analysis showing the heat map of top 50 features for each phenotype based on metastasis of PC/PG cases in (a) TCGA and (b) COMETE cohort. PC: pheochromocytoma; PG: paraganglioma; M: malignant; B: benign.

**Figure 5:**
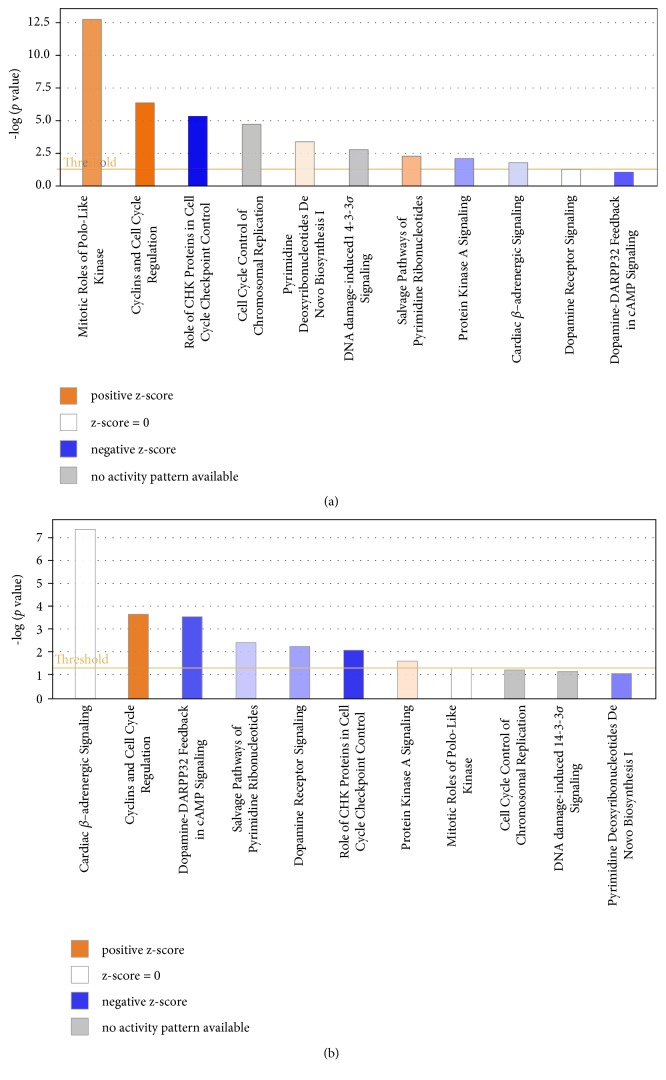
Ingenuity Canonical Analysis showing 11 pathways harboring the corresponding genes in the transcriptome analysis to differentiate malignant PC/PG in (a) TCGA and (b) COMETE cohort. PC: pheochromocytoma; PG: paraganglioma.

**Figure 6:**
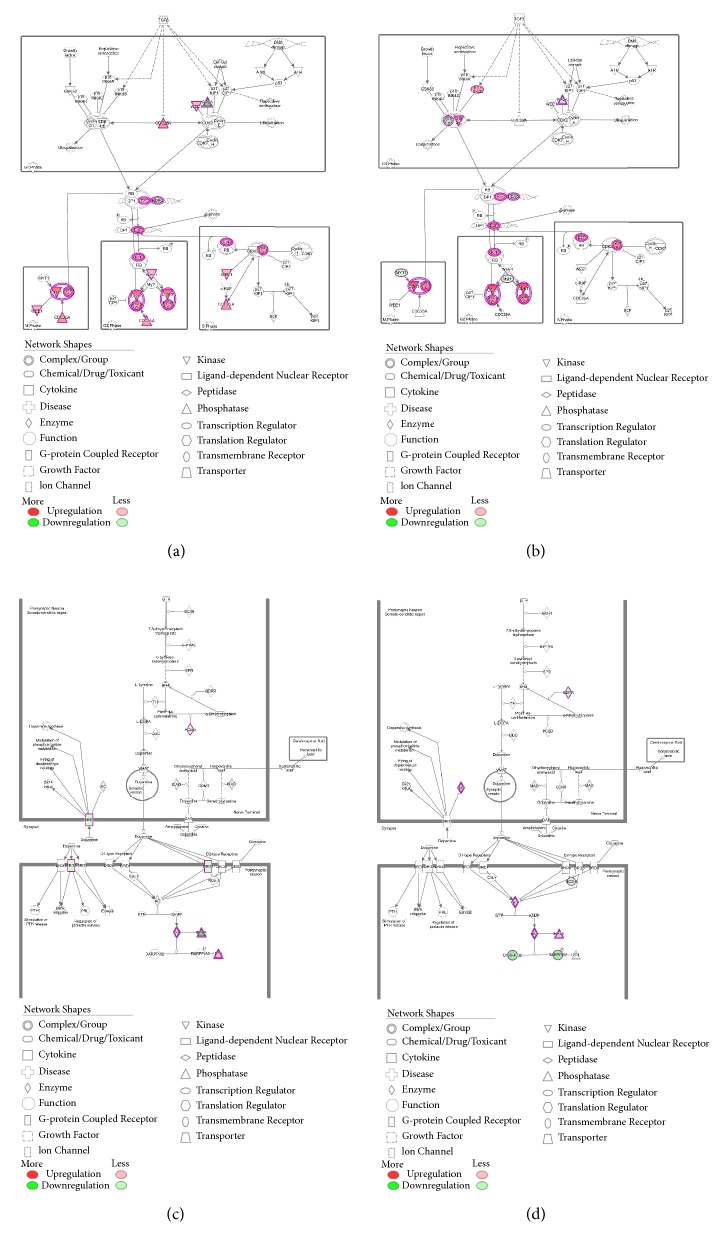
Up/downregulated genes in enriched pathways: cyclins and cell cycle of (a) TCGA and (b) COMETE cohort and dopaminergic synapse of (c) TCGA and (d) COMETE cohort.

**Figure 7:**
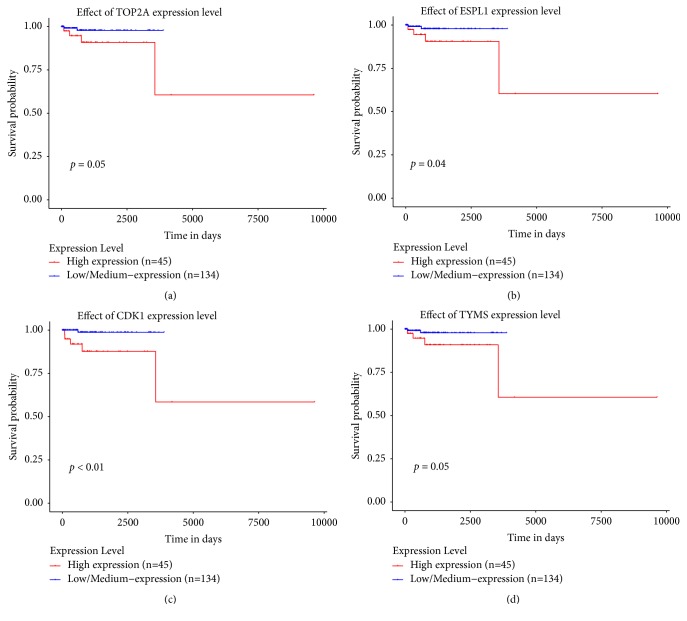
When the identified genes were analyzed in UALCAN, overall survival rate in TCGA was considerably significantly with four genes: (a)* TOP2A*, (b)* ESPL1*, (c)* CDK1*, and (d)* TYMS*.

In TCGA, 6,056 out of 20,531 genes were excluded because at least one sample scored zero value. Based on a comparative analysis of 14,475 genes, 367 upregulated genes were identified while 282 genes were downregulated. A total of 39,534 probes were used to analyze COMETE cohort. Results showed an upregulation of 558 probes and downregulation of 1,132 probes. In both data, the quality control was performed for each gene or probe. The gene expression was analyzed in various ways. Functional annotation analysis was used for gene set enrichment (Figure 4). The top 50 features identified in Gene Set Enrichment Analysis are listed in Supplemental Table 1. Commonly enriched pathways in malignant PC/PG were linked to significant mitosis, metabolic alteration, and cancer signaling (Figure 5). Hierarchical clustering analysis was performed by Euclidean distance and complete linkage. The common up/downregulated differentially expressed genes (DEGs) were extracted from TCGA and COMETE cohort (Supplemental Table 2). The 11 up/downregulated pathways harbored over/underexpressed genes (Table 3). The cyclin and cell cycle regulation as well as the dopamine receptor signaling representing pathways in TCGA and COMETE cohort were displayed in Figure 6. The seven common genes identified were validated in the Dutch cohort (GSE67066). As potential biomarkers, the seven genes in the current study are presented in bold (Supplemental Table 3). Among the seven common genes identified, the overall survival rate in TCGA was significantly correlated with the expression of four genes (*TOP2A*,* ESPL1*,* CDK1*, and* TYMS*) (Figure 7).

## 4. Discussion

Currently few reliable histopathological criteria predict malignant behavior in PC/PG. Studies to date reported prognostic factors for malignant PC/PG including older age, greater tumor size, extra-adrenal location, elevated dopamine, and synchronous metastasis [13, 14, 19, 25–27]. In the present study, the possible clinical risk factors included dopamine secretion, PG, and greater tumor size. These observations were generally consistent with previous studies. Differences in genomic expression of malignant PC/PG were investigated using data derived from TCGA and COMETE cohort to predict the clinical prognosis. The 11 up/downregulated pathways in malignant PC/PG were significantly associated with the clinical phenotype of increased tumor size and dopamine secretion. Six upregulated and one downregulated genes were significantly enriched in functional annotation pathways. In PC/PG transformed to malignant types, cellular or nuclear proliferation, signaling network, and metabolic changes were essential processes linked to cancer progression. Among the seven common genes, four genes were considerably correlated with overall survival rate.

Grading for adrenal pheochromocytoma and paraganglioma (GAPP) and pheochromocytoma of the adrenal gland scaled score (PASS) have been developed to predict malignancy based on histopathology [28–30]. These two risk stratification systems show several common features, including high cellularity, tumor necrosis, vascular or capsular invasion, and large nest. The recurrent themes carry implied validity. However, these systems are limited intrinsically or due to the absence of consistent validation [28, 30].

In one study, 58 pheochromocytoma samples were analyzed to distinguish malignant samples [31]. Based on lymph node or distant metastasis, 13 samples were classified as malignant. Genome-wide expression profiling was used to select 10 genes among 109 DEGs which were selected. The present study had a similar focus. However, the current study was designed in response to the challenges documented in the 8th edition of the AJCC staging system. Malignant tumors may not be associated with local invasion or locoregional lymph node metastases. Therefore, a stricter definition of malignancy was used to classify patients with PC/PG. Data derived from the two public databases were used for consistency. Even in functionally enriched pathways, each cascade was investigated for common genes. The 11 functional pathways were presumably transformed to malignancy, and included six upregulated and one downregulated genes.

The current results were based on accumulated biological information. These genes were validated in TCGA and COMETE cohort. In other studies, the tumorigenesis of PC/PG was explained via alteration in the three representative molecular signaling pathways including pseudohypoxia signaling, kinase signaling, and* WNT* signaling [18, 20, 21, 32, 33]. Activation of effector molecules in pseudohypoxia signaling is triggered by genetic mutations involving the degradation of hypoxia‐inducible factor 1/2*α* or Krebs cycle function, such as succinate dehydrogenase genes (*SDHx*),* VHL*, or* EPAS1* [34]. These changes suggest increased levels of angiogenesis and enable hematogenous dissemination [35, 36]. Hyperactive kinase signaling is induced by mutations involving genes associated with mitogen‐activated protein kinase, such as tumor suppressor genes (*NF1, TMEM127, MAX,* and* KIF1Bβ*),* RET,* or* HRAS*, which promoted growth independence of extracellular signals [35, 37, 38]. Additionally, upregulated* WNT* signaling has been recently reported in genetic alterations of* MAML3* and* CSDE1* [20].

In the present study, DEGs were enriched in 11 presumptive pathways that interacted closely with the three known molecular pathways mentioned above. Further, the 11 presumptive pathways mediated malignant transformation, including cellular or nuclear proliferation, signaling, and metabolic changes predicting cancer progression. However, whether these pathways directly mediated metastasis or the by-product circuits remains to be investigated.

Among the 11 pathways, six common genes (*TOP2A, ESPL1, CDK1, TYMS, CDT1,* and* CCNA2*) were differentially overexpressed, and one common gene (*PRKACB*) was underexpressed in malignant PC/PG. These genes play a role in cell cycle, cell signaling, and tumor metabolism of malignant PC/PG as well as in other cancers. These six overexpressed genes play a critical role in signaling pathways or cell proliferation, which can increase the tumor size in malignant PC/PG as shown in Table 1. The underexpressed gene was correlated with signaling pathways or metabolic changes, which may lead to differential catecholamine secretion as shown in Table 2. Abnormal* TOP2A* overexpression leads to chromosomal instability.* ESPL1* plays a pivotal role in chromatid separation. The overexpression of* CDK1, TYMS, CDT1,* and* CCNA2* and the underexpression of* PRKACB* were associated with tumorigenesis, defective cell signaling, or aberrant metabolism in other cancers [39]. These genes served as prognostic genes for malignant PC/PG in this study.

Germline mutations have been reported in 20-41% cases of PC/PG [3]. Comprehensive analyses have implicated germline mutations involving* SDHB*,* FH*,* MAX*, and* SLC25A11* in the origin and development of malignant PC/PG [40–43]. It is generally recognized that PC/PG carrying germline mutations of* SDHB* show a higher rate of metastasis [44]. The* SDHB* mutation in Krebs cycle impairs glucose metabolism, leading to inhibition of 2-oxoglutarate-dependent histone and DNA demethylase enzymes. The mutation rate and the spectrum of malignant PC/PG were not comparable in these different cohorts. The germline mutation of* SDHB* was significantly susceptible to malignancy in TCGA and COMETE cohort (*p* = 0.0049 and* p* < 0.001, respectively). Mutations involving* SDHx* or* FH* lead to DNA hypermethylation, explaining both the tumor-suppressive role of these genes and the phenotypic characteristics [45]. In particular, the malignancy of* SDHB* mutation is attributed to severe epigenetic silencing of genes involved in cell differentiation and epithelial-to-mesenchymal transition.* RDBP* hypermethylation may alter transcriptional networks involving apoptosis, invasion, and maintenance of DNA integrity [46].

To the best of our knowledge, this study is the first transcriptome analysis identifying prognostic genes for malignant PC/PG defined in the 8th edition of the AJCC staging system. We identified potential pathways leading to malignant transformation of PC/PG and subsequently up/downregulated genes in malignant PC/PG. Specific genes in the current study may be used for the development of gene expression classifier [47]. The development of gene expression classifier is expected to improve the diagnostic accuracy and treatment decision. Novel molecular therapeutics can be developed based on the results of the current study.

The current study has a few limitations. First, no comprehensive analysis of microRNA, DNA methylation, copy-number variation, mutation, or protein expression was carried out to establish the complete signature of malignant PC/PG. Second, only two proven databases were used in the present study. Identification of databases for genomic analysis is difficult because PC/PG is a rare disease. Third, as a general limitation of public data analysis, the present study was based on limited data provided.

In conclusion, data from the TCGA database and the COMETE cohort showed differences in mRNA expression between malignant and benign PC/PG. Improved recognition of prognostic genes based on our analyses will facilitate appropriate diagnosis and treatment of malignant PC/PG.

## Figures and Tables

**Table 1:** Clinical demographics of malignant and benign PC/PG cases obtained from the TCGA database and the COMETE cohort.

Characteristic	Malignant (n = 30)	Benign (n = 199)	*p* value
Age (years), mean ± SD	38.9 ± 14.4	43.2 ± 15.7	0.140
Gender, n (%)	30	199	0.432
Male	15 (50.0)	83 (51.1)	
Female	15 (50.0)	116 (48.9)	
Race, n (%)	11	46	0.497
White	10 (90.9)	36 (78.2)	
African-American	1 (9.1)	5 (10.9)	
Asia	0 (0)	5 (10.9)	
Laterality, n (%)	6	41	0.749
Right	2 (33.3)	19 (46.4)	
Left	4 (66.7)	21 (51.2)	
Bilaterality	0 (0)	1 (2.4)	
Diagnosis, n (%)	30	199	<0.001
PC	15 (50.0)	177 (87.2)	
PG	15 (50.0)	22 (12.8)	
Size (mm), mean ± SD	73.4 ± 28.1	46.8 ± 20.2	<0.001
Follow-up (days), mean ± SD	2460.2 ± 2658.9	1617.6 ± 867.7	0.320

PC: pheochromocytoma; PG: paraganglioma.

**Table 2:** Catecholamine secretion by malignant and benign PC/PG cases derived from TCGA.

Characteristic	Malignant (n = 9)	Benign (n = 40)	*p* value
Biochemical testing			
Normetanephrine	6	34	0.336
Norepinephrine	5	24	1.000
Epinephrine	1	15	0.238
Metanephrine	0	21	0.006
Methoxytyramine	1	0	0.184
Dopamine	4	5	0.046

PC: pheochromocytoma; PG: paraganglioma.

**Table 3:** Ingenuity Canonical Pathways and the corresponding genes in the transcriptome analysis differentiating malignant PC/PG cases in TCGA and COMETE cohort.

Ingenuity Canonical Pathways	Regulation	
	*Up*	*Down*
Cyclins and Cell Cycle Regulation	*CCNA2, CDK1 *	
Dopamine-DARPP32 Feedback in cAMP Signaling		*PRKACB*
Mitotic Roles of Polo-Like Kinase	*CDK1, ESPL1*	
Cell Cycle Control of Chromosomal Replication	*CDK1, CDT1, TOP2A*	
Cardiac *β*-adrenergic Signaling		*PRKACB*
Dopamine Receptor Signaling		*PRKACB*
Role of CHK Proteins in Cell Cycle Checkpoint Control	*CDK1*	
Pyrimidine Deoxyribonucleotides De Novo Biosynthesis I	*TYMS*	
Salvage Pathways of Pyrimidine Ribonucleotides	*CDK1*	
Protein Kinase A Signaling		*PRKACB*
DNA damage-induced 14-3-3*σ* Signaling	*CDK1*	

PC: pheochromocytoma; PG: paraganglioma.

## Data Availability

The data used to support the findings of this study are available from the corresponding author upon request.

## Supplementary Materials

Supplementary Materials 
* Supplemental Table 1:* the top 50 features of Gene Set Enrichment Analysis based on metastasis in pheochromocytoma/paraganglioma from TCGA and COMETE cohort.* Supplemental Table 2:* the common differentially expressed (up/downregulated) genes (DEGs) extracted from TCGA and COMETE cohort.* Supplemental Table 3:* the 34 common differentially expressed (up/downregulated) genes (DEGs) validated in GSE67066 cohort.
