# Supplementary material for: Transcriptome Analysis Reveals Significant Differences in Gene Expression of Malignant Pheochromocytoma or Paraganglioma
Source: Int J Endocrinol. 2019 May 8;2019:7014240. doi: 10.1155/2019/7014240 (PMC6530119; doi:10.1155/2019/7014240)
Supplement: Supplementary Materials — Supplemental Table 1: the top 50 features of Gene Set Enrichment Analysis based on metastasis in pheochromocytoma/paraganglioma from TCGA and COMETE cohort. Supplemental Table 2: the common differentially expressed (up/downregulated) genes (DEGs) extracted from TCGA and COMETE cohort. Supplemental Table 3: the 34 common differentially expressed (up/downregulated) genes (DEGs) validated in GSE67066 cohort. [file 7014240.f1.docx]

**Supplementary TABLE 1:** The top 50 features of Gene Set Enrichment Analysis based on metastasis in pheochromocytoma/paraganglioma from TCGA and COMETE cohort.

| TCGA | | COMETE cohort | |
| --- | --- | --- | --- |
| *COCH* | coagulation factor C homolog, cochlin | *TWIST1* | twist homolog 1 (acrocephalosyndactyly 3 |
| *PRKCB* |  | *TOP2A* | topoisomerase (DNA) II alpha |
| *ST8SIA2* | ST8 alpha-N-acetyl-neuraminide alpha-2,8-sialyltransferase 2 | *COX4I2* | cytochrome c oxidase subunit IV isoform 2 |
| *MYBL2* | v-myb myeloblastosis viral oncogene homolog (avian)-like 2 | *CNTN4* | contactin 4 |
| *BUB1* | BUB1 budding uninhibited by benzimidazoles 1 homolog | *NPNT* | nephronectin |
| *DLK2* |  | *LAYN* | layilin |
| *PMAIP1* | phorbol-12-myristate-13-acetate-induced protein 1 | *KCNE3* | potassium voltage-gated channel, Isk-related family, member 3 |
| *FAM83H* | family with sequence similarity 83, member H | *PKIB* | protein kinase (cAMP-dependent, catalytic) inhibitor beta |
| *RYR2* | ryanodine receptor 2 | *ANLN* | anillin, actin binding protein |
| *BUB1B* | BUB1 budding uninhibited by benzimidazoles 1 homolog beta | *GJA7* | gap junction protein, alpha (connexin 45) |
| *LRRN1* | leucine rich repeat neuronal 1 | *TMTC4* | transmembrane and tetratricopeptide repeat containing 4 |
| *KIF23* | kinesin family member 23 | *RHOU* | ras homolog gene family, member U |
| *ASPM* | asp (abnormal spindle) homolog, microcephaly associated | *HK2* | hexokinase 2 |
| *TOP2A* | topoisomerase (DNA) II alpha | *CDK1* | cell division cycle 2, G1 to S and G2 to M |
| *TTK* | TTK protein kinase | *NTNG2* | netrin G2 |
| *DGCR5* | DiGeorge syndrome critical region gene 5 | *SOX11* | SRY (sex determining region Y)-box 11 |
| *TMTC4* | transmembrane and tetratricopeptide repeat containing 4 | *BCL11B* | B-cell CLL/lymphoma 11B (zinc finger protein) |
| *DLGAP5* |  | *IGFBP3* | insulin-like growth factor binding protein 3 |
| *MELK* | maternal embryonic leucine zipper kinase | *CXCR7* | chemokine (C-X-C motif) receptor 7 |
| *IQGAP3* | IQ motif containing GTPase activating protein 3 | *SLC16A10* | solute carrier family 16, member 10 |
| *ANLN* | anillin, actin binding protein | *KIAA1199* |  |
| *DIAPH3* | diaphanous homolog 3 | *CNTNAP4* | contactin associated protein-like 4 |
| *SATB2* | SATB family member 2 | *HOXA9* | homeobox A9 |
| *KIF14* | kinesin family member 14 | *CCL18* | chemokine (C-C motif) ligand 18 |
| *TROAP* | trophinin associated protein (tastin) | *BCAT1* | branched chain aminotransferase 1, cytosolic |
| *ETV4* | ets variant gene 4 | *ELAVL3* | ELAV-like 3 |
| *CENPF* | centromere protein F, 350/400ka (mitosin) | *HOXD10* | homeobox D10 |
| *MEX3A* |  | *CTHRC1* | collagen triple helix repeat containing 1 |
| *ELAVL3* | ELAV-like 3 | *ZNF300* | zinc finger protein 300 |
| *BIRC5* | baculoviral IAP repeat-containing 5 (survivin) | *NLF1* | - |
| *DSP* | desmoplakin | *STC1* | stanniocalcin 1 |
| *ESPL1* | extra spindle poles like 1 | *FAM72A* |  |
| *NCAPH* | non-SMC condensin I complex, subunit H | *ADAMTS5* | ADAM metallopeptidase with thrombospondin type 1 motif, 5 |
| *UBE2C* | ubiquitin-conjugating enzyme E2C | *BVES* | blood vessel epicardial substance |
| *KIF20A* | kinesin family member 20A | *BCL11A* | B-cell CLL/lymphoma 11A |
| *HJURP* |  | *FAM83D* | family with sequence similarity 83, member D |
| *KIF18A* | kinesin family member 18A | *FLT1* | fms-related tyrosine kinase 1 |
| *NEK2* | NIMA (never in mitosis gene a)-related kinase 2 | *UHRF1* | ubiquitin-like, containing PHD and RING finger domains, 1 |
| *CDK1* |  | *LARGE* | like-glycosyltransferase |
| *PIMREG* |  | *C2ORF10* | chromosome 2 open reading frame 10 |
| *AURKB* | aurora kinase B | *THBS1* | thrombospondin 1 |
| *KCNT2* | potassium channel, subfamily T, member 2 | *COL14A1* | collagen, type XIV, alpha 1 (undulin) |
| *DTL* | denticleless homolog | *SMPDL3A* | sphingomyelin phosphodiesterase, acid-like 3A |
| *CDC45* |  | *SOCS3* | suppressor of cytokine signaling 3 |
| *RRM2* | ribonucleotide reductase M2 polypeptide | *RRM2* | ribonucleotide reductase M2 polypeptide |
| *GRIK4* | glutamate receptor, ionotropic, kainate 4 | *PXDN* | peroxidasin homolog |
| *CNTN4* | contactin 4 | *CCNA2* | cyclin A2 |
| *TYMS* | thymidylate synthetase | *TYMS* | thymidylate synthetase |
| *CDT1* | chromatin licensing and DNA replication factor 1 | *CDT1* | chromatin licensing and DNA replication factor 1 |
| *CCNA2* | cyclin A2 | *ESPL1* | extra spindle poles like 1 |

(Continued)

| *ARHGAP36* |  | *RP13-102H20.1* | - |
| --- | --- | --- | --- |
| *PENK* | proenkephalin | *GABRG2* | gamma-aminobutyric acid (GABA) A receptor, gamma 2 |
| *FAM19A3* | family with sequence similarity 19, member A3 | *PENK* | proenkephalin |
| *PLCH2* | phospholipase C, eta 2 | *CNTN3* | contactin 3 (plasmacytoma associated) |
| *SLC6A2* | solute carrier family 6, member 2 | *IGSF4D* | immunoglobulin superfamily, member 4D |
| *PPM1J* | protein phosphatase 1J (PP2C domain containing) | *DNAJA4* | DnaJ (Hsp40) homolog, subfamily A, member 4 |
| *PNMT* | phenylethanolamine N-methyltransferase | *SST* | somatostatin |
| *RET* | ret proto-oncogene | *SYT13* | synaptotagmin XIII |
| *HAPLN4* | hyaluronan and proteoglycan link protein 4 | *JAKMIP1* | janus kinase and microtubule interacting protein 1 |
| *NTRK1* | neurotrophic tyrosine kinase, receptor, type 1 | *FLJ39822* | - |
| *DLX1* | distal-less homeobox 1 | *RET* | ret proto-oncogene |
| *NPY* | neuropeptide Y | *GPX3* | glutathione peroxidase 3 (plasma) |
| *KRT19* | keratin 19 | *FLJ30428* | - |
| *ZFR2* |  | *CYP11B1* | cytochrome P450, family 11, subfamily B, polypeptide 1 |
| *NTNG1* | netrin G1 | *GNG8* | guanine nucleotide binding protein , gamma 8 |
| *ADGRA1* |  | *C1ORF173* | chromosome 1 open reading frame 173 |
| *IGSF10* | immunoglobulin superfamily, member 10 | *PON3* | paraoxonase 3 |
| *ARC* | activity-regulated cytoskeleton-associated protein | *SPOCK3* | sparc/osteonectin, cwcv and kazal-like domains proteoglycan 3 |
| *IGSF1* | immunoglobulin superfamily, member 1 | *PDE8B* | phosphodiesterase 8B |
| *DBH* | dopamine beta-hydroxylase | *SLC6A2* | solute carrier family 6, member 2 |
| *CNTFR* | ciliary neurotrophic factor receptor | *GPR176* | G protein-coupled receptor 176 |
| *KIRREL3* | kin of IRRE like 3 | *NGB* | neuroglobin |
| *SULT4A1* | sulfotransferase family 4A, member 1 | *RFXDC1* | regulatory factor X domain containing 1 |
| *PRDM8* | PR domain containing 8 | *CDKN1C* | cyclin-dependent kinase inhibitor 1C |
| *VSTM2L* |  | *RAB27B* | RAB27B, member RAS oncogene family |
| *NEFH* | neurofilament, heavy polypeptide | *NEFH* | neurofilament, heavy polypeptide |
| *FAM189A1* |  | *CYP21A2* | cytochrome P450, family 21, subfamily A, polypeptide 2 |
| *SHISA9* |  | *KRT222P* | keratin 222 pseudogene |
| *TM6SF2* | transmembrane 6 superfamily member 2 | *ANKRD43* | ankyrin repeat domain 43 |
| *CALY* |  | *GAL* | galanin |
| *CHRNB4* | cholinergic receptor, nicotinic, beta 4 | *MGC33846* | - |
| *KCNJ5* | potassium inwardly-rectifying channel, subfamily J, member 5 | *GPR123* | G protein-coupled receptor 123 |
| *SPOCK3* | sparc/osteonectin, cwcv and kazal-like domains proteoglycan 3 | *IGSF10* | immunoglobulin superfamily, member 10 |
| *C2CD4B* |  | *LOC285878* | - |
| *BAIAP3* | BAI1-associated protein 3 | *HAPLN4* | hyaluronan and proteoglycan link protein 4 |
| *HCN1* |  | *PPP1R1B* | protein phosphatase 1, regulatory (inhibitor) subunit 1B |
| *PHYHIP* | phytanoyl-CoA 2-hydroxylase interacting protein | *NPAL2* | NIPA-like domain containing 2 |
| *PLXNA4* |  | *HSPB6* | heat shock protein, alpha-crystallin-related, B6 |
| *RPH3A* | rabphilin 3A homolog | *PLCXD3* |  |
| *ZCCHC12* | zinc finger, CCHC domain containing 12 | *AMIGO2* | adhesion molecule with Ig-like domain 2 |
| *EPHX2* | epoxide hydrolase 2, cytoplasmic | *ZCCHC12* | zinc finger, CCHC domain containing 12 |
| *DNAH5* | dynein, axonemal, heavy chain 5 | *MGST1* | microsomal glutathione S-transferase 1 |
| *PCDH10* | protocadherin 10 | *DMRTC1* |  |
| *LRRC10B* |  | *PRLR* | prolactin receptor |
| *STAR* | steroidogenic acute regulator | *LRRC4C* | leucine rich repeat containing 4C |
| *RPS28* | ribosomal protein S28 | *C1ORF36* | chromosome 1 open reading frame 36 |
| *TFPI2* | tissue factor pathway inhibitor 2 | *NRXN3* | neurexin 3 |
| *IGSF11* | immunoglobulin superfamily, member 11 | *DKK3* | dickkopf homolog 3 |
| *TCEAL5* | transcription elongation factor A (SII)-like 5 | *TMIE* | transmembrane inner ear |
| *PRKACB* | protein kinase, cAMP-dependent, catalytic, beta | *PRKACB* | protein kinase, cAMP-dependent, catalytic, beta |

**Supplementary TABLE 2:** The common differentially expressed (up/down-regulated) genes (DEGs) extracted from TCGA and COMETE cohort.

| Gene | Description | Up/down |
| --- | --- | --- |
| *BUB1* | BUB1 mitotic checkpoint serine/threonine kinase |  |
| *PMAIP1* | phorbol-12-myristate-13-acetate-induced protein 1 |  |
| *CNTN4* | contactin 4 |  |
| *RYR2* | ryanodine receptor 2 |  |
| *TOP2A* | topoisomerase (DNA) II alpha |  |
| *IQGAP3* | IQ motif containing GTPase activating protein 3 |  |
| *DIAPH3* | diaphanous related formin 3 |  |
| *BIRC5* | baculoviral IAP repeat containing 5 |  |
| *ESPL1* | extra spindle pole bodies like 1, separase |  |
| *CDK1* | cyclin dependent kinase 1 |  |
| *RRM2* | ribonucleotide reductase regulatory subunit M2 |  |
| *MKI67* | marker of proliferation Ki-67 |  |
| *BRIP1* | BRCA1 interacting protein C-terminal helicase 1 |  |
| *TK1* | thymidine kinase 1 |  |
| *TYMS* | thymidylate synthetase |  |
| *CDT1* | chromatin licensing and DNA replication factor 1 |  |
| *ELOVL7* | ELOVL fatty acid elongase 7 |  |
| *CCNA2* | cyclin A2 |  |
| *ELOVL2* | ELOVL fatty acid elongase 2 |  |
| *RMI2* | RecQ mediated genome instability 2 |  |
| *FANCI* | Fanconi anemia complementation group I |  |
| *CCNB1* | cyclin B1 |  |
| *ITGAV* | integrin subunit alpha V |  |
| *TSC2* | tuberous sclerosis 2 |  |
| *ACOT7* | acyl-CoA thioesterase 7 |  |
| *PRKACB* | protein kinase cAMP-activated catalytic subunit beta |  |
| *CDKN1C* | cyclin dependent kinase inhibitor 1C |  |
| *GPX3* | glutathione peroxidase 3 |  |
| *HDAC11* | histone deacetylase 11 |  |
| *HIST1H4H* | histone cluster 1 H4 family member h |  |
| *HIST1H2AC* | histone cluster 1 H2A family member c |  |
| *KCNJ5* | potassium voltage-gated channel subfamily J member 5 |  |
| *NTNG1* | netrin G1 |  |
| *RET* | ret proto-oncogene |  |

**Supplementary TABLE 3:** The 34 common differentially expressed (up/down-regulated) genes (DEGs) validated in GSE67066 cohort.

| Gene | Description | *p* value | log (FC) |
| --- | --- | --- | --- |
| *CNTN4* | contactin 4 | 0.000015 | 2.316018 |
| *TOP2A* | topoisomerase (DNA) II alpha | 0.001990 | 1.333828 |
| *CDK1* | cyclin dependent kinase 1 | 0.004400 | 1.164186 |
| *RRM2* | ribonucleotide reductase regulatory subunit M2 | 0.005610 | 1.156551 |
| *ELOVL7* | ELOVL fatty acid elongase 7 | 0.030400 | 1.087779 |
| *BIRC5* | baculoviral IAP repeat containing 5 | 0.001430 | 0.965904 |
| *BUB1* | mitotic checkpoint serine/threonine kinase | 0.003520 | 0.936124 |
| *RYR2* | ryanodine receptor 2 | 0.019000 | 0.877898 |
| *CCNB1* | cyclin B1 | 0.027000 | 0.855084 |
| *TYMS* | thymidylate synthetase | 0.042600 | 0.737806 |
| *FANCI* | Fanconi anemia complementation group I | 0.015300 | 0.710895 |
| *PMAIP1* | phorbol-12-myristate-13-acetate-induced protein 1 | 0.240000 | 0.692439 |
| *CCNA2* | cyclin A2 | 0.033700 | 0.620342 |
| *IQGAP3* | IQ motif containing GTPase activating protein 3 | 0.024400 | 0.590179 |
| *MKI67* | marker of proliferation Ki-67 | 0.011100 | 0.525830 |
| *RMI2* | RecQ mediated genome instability 2 | 0.011800 | 0.432221 |
| *CDT1* | chromatin licensing and DNA replication factor 1 | 0.012000 | 0.385388 |
| *DIAPH3* | diaphanous related formin 3 | 0.021200 | 0.372634 |
| *TK1* | thymidine kinase 1 | 0.008570 | 0.345759 |
| *ESPL1* | extra spindle pole bodies like 1, separase | 0.027500 | 0.338947 |
| *ITGAV* | integrin subunit alpha V | 0.237000 | 0.326175 |
| *BRIP1* | BRCA1 interacting protein C-terminal helicase 1 | 0.020000 | 0.241226 |
| *HIST1H4H* | histone cluster 1, H4h | 0.512000 | 0.239268 |
| *HIST1H2AC* | histone cluster 1, H2ac | 0.780000 | 0.123353 |
| *ELOVL2* | ELOVL fatty acid elongase 2 | 0.188000 | -0.170116 |
| *CDKN1C* | cyclin dependent kinase inhibitor 1C | 0.534000 | -0.288124 |
| *KCNJ5* | potassium voltage-gated channel subfamily J member 5 | 0.011000 | -0.361025 |
| *HDAC11* | histone deacetylase 11 | 0.000121 | -0.392601 |
| *ACOT7* | acyl-CoA thioesterase 7 | 0.030200 | -0.580283 |
| *TSC2* | tuberous sclerosis 2 | 0.047000 | -0.628722 |
| *PRKACB* | protein kinase cAMP-activated catalytic subunit beta | 0.041600 | -0.744727 |
| *NTNG1* | netrin G1 | 0.012600 | -1.013644 |
| *RET* | ret proto-oncogene | 0.088900 | -1.384179 |
| *GPX3* | glutathione peroxidase 3 | 0.005200 | -1.740770 |

FC: fold change.
